# Letter from the Editor in Chief

**DOI:** 10.19102/icrm.2022.13103

**Published:** 2022-10-15

**Authors:** Moussa Mansour


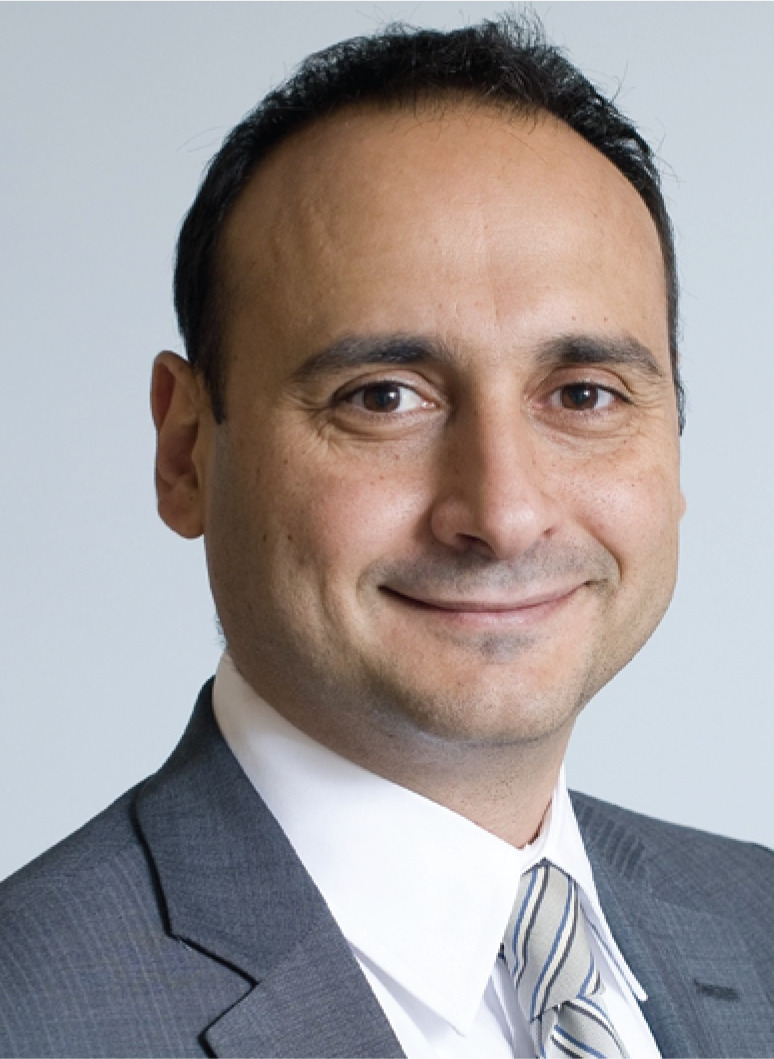


Dear readers,

Atrial fibrillation (AF) affects a large number of people in the United States, and it is estimated that 12–16 million people nationwide will have AF by the year 2050.^1^ This will result in a major clinical and economic burden that will add significant stress to the health care system.

Catheter ablation has been demonstrated to improve the outcome of patients with AF and is considered a first-line therapy in many situations. As a result, the use of catheter ablation is rapidly growing in the United States at an estimated annual rate of 20%. Despite this growth, however, only a small portion of patients with AF have access to the ablation procedure. One factor limiting the wider spread of this therapeutic intervention is the challenge of developing the infrastructure that allows for AF ablation to be performed safely and successfully.

In this issue of *The Journal of Innovations in Cardiac Rhythm Management*, I would like to highlight the article titled “Designing an Efficient and Quality-focused Integrated Atrial Fibrillation Care Center” by Silverstein et al.^2^ In it, the authors describe their recipe for a successful AF center and, more importantly, discuss the reproducibility of their development process at other centers. The cornerstone of their successful operation is a fully integrated system that is not limited only to the ablation procedure; instead, it also incorporates emergency department care, pre-procedural planning, collaboration with anesthesia, and post-procedural follow-up and data collection.

An integrated care model for AF is necessary to improve the outcome of ablation, reduce the rate of redo procedures, lower complications, and subsequently reduce the cost of care for AF. Replicating such a model will grant many more patients access to ablation.

I hope that you enjoy reading this issue of *The Journal of Innovations in Cardiac Rhythm Management*.


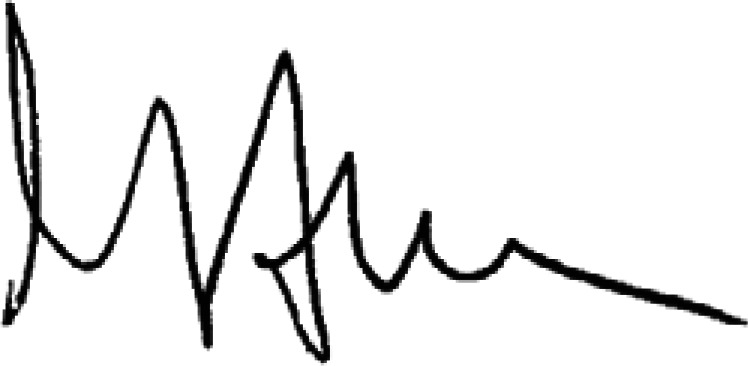


Sincerely,

Moussa Mansour, md, fhrs, facc

Editor in Chief

*The Journal of Innovations in Cardiac Rhythm Management*

MMansour@InnovationsInCRM.com

Director, Atrial Fibrillation Program

Jeremy Ruskin and Dan Starks Endowed Chair in Cardiology

Massachusetts General Hospital

Boston, MA 02114
